# The Relationship Between BMI and Postoperative Complications Among Colorectal Cancer Patients Undergoing Surgery

**DOI:** 10.7759/cureus.48715

**Published:** 2023-11-13

**Authors:** Ayyob Alqarni, Fahad Aljehaiman, Saad A Almousa, Sundos A Almarshad, Fahad K Alrzouq

**Affiliations:** 1 Department of General and Colorectal Surgery, King Abdulaziz Medical City, Riyadh, SAU; 2 Collage of Medicine, King Saud Bin Abdulaziz University for Health Sciences, Riyadh, SAU; 3 College of Medicine, King Saud Bin Abdulaziz University for Health Sciences, Riyadh, SAU; 4 Department of Research, King Abdullah International Medical Research Center, Riyadh, SAU

**Keywords:** bmi, mortality, length of hospital stay, postoperative complications, colorectal cancer

## Abstract

Introduction: Colorectal cancer (CRC) is common worldwide, and surgery is one of the main treatments. Postoperative complications are a concern. The primary objective of this study is to determine whether elevated body mass index (BMI), the presence of comorbidities, tumor characteristics, and the type of surgery are associated with an increased risk of postoperative complications such as wound infections, pulmonary complications, anastomotic leak, venous thromboembolism (VTE), bowel obstruction, and incisional hernia. The secondary objective is to describe the characteristics of colorectal cancer patients with different BMI groups.

Methodology: A retrospective cohort study was conducted using a non-probability sampling technique at a tertiary National Guard Hospital in Riyadh, Saudi Arabia. This study involved 122 patients aged 18 years or more who underwent elective or emergency surgery for colorectal cancer between the years 2015 and 2022. Data analysis was performed using Statistical Package for Social Sciences (SPSS) version 27 (IBM SPSS Statistics, Armonk, NY). Descriptive statistics (mean and standard deviation) were used for quantitative variables, while qualitative variables were presented as percentages and frequencies. Non-parametric tests were applied to compare qualitative variables. Quantitative variables were analyzed using the analysis of variance (ANOVA) test. Significance was established at a p-value of 0.05. Ethical considerations were followed throughout the study. Prior to conducting the study, ethical approval was obtained from the Institutional Review Board of King Abdullah International Medical Research Center (KAIMRC) (approval number: IRB/1598/23).

Results: High BMI scores were observed in patients with postoperative complications. A statistically significant variation in BMI scores (p-value < 0.05) was found between patients with complications and without complications. This observation suggests that factors beyond excessive body weight might contribute to the onset of postoperative complications. Moreover, elevated BMI scores were more prevalent in males and were associated with reduced hemoglobin (Hgb) levels, underscoring the potential influence of physiological variables on the emergence of postoperative complications (p-value < 0.05). Tumors located in the rectum or rectosigmoid regions, as well as partial colectomy procedures, exhibited a higher risk of postoperative complications (p-value < 0.05). However, no significant connections were identified between the presence of comorbidities and the occurrence of postoperative complications (p-value > 0.05).

Conclusion: This study highlights the impact of BMI on postoperative outcomes in colorectal cancer patients. Higher BMI was associated with unfavorable postoperative outcomes, such as an increased risk of VTE and fluid collection. However, no significant differences in mortality rates or length of hospital stay (LOS) were observed across various BMI categories. Factors beyond BMI, including tumor characteristics, the type of surgical intervention, and preoperative care, play a significant role in determining postoperative outcomes. Therefore, it is essential to adopt a comprehensive approach that considers multiple factors when managing postoperative complications in colorectal cancer patients, particularly those with higher BMI.

## Introduction

Colorectal cancer (CRC) is a common type of malignancy worldwide, with an estimated 1.9 million new cases and 935,000 deaths in 2020 alone [1]. Surgical resection remains the primary curative treatment option for patients with localized colorectal cancer. However, postoperative complications remain a significant concern. Previous studies have shown that elevated body mass index (BMI) is associated with an increased risk of postoperative complications such as wound infections [2,3].

BMI is a simple anthropometric measure that quantifies a person’s weight relative to their height. It has been established as an indicator of obesity and is used as a screening tool for identifying individuals at risk of developing obesity-related health problems. Obesity has been recognized as a significant public health issue in developed countries, with its prevalence increasing steadily over the past few decades. A study conducted in the United States demonstrated that approximately 42% of adults are categorized as obese based on BMI [4]. The association between BMI and postoperative complications has been researched extensively, and several studies have provided evidence supporting this relationship [5-7]. While the association between BMI and postoperative complications has been established in the general surgical population, the relationship between BMI and postoperative complications among colorectal cancer patients undergoing surgery is not yet fully elucidated. Some studies have reported an increased risk of postoperative complications with increasing BMI in this population [8], while others found no significant association [9].

Furthermore, the mechanisms that underlie the association between obesity and colorectal cancer mortality are unclear, but several studies have addressed possible mechanisms, mainly related to obesity-related hormonal changes. Obesity is associated with elevations in insulin, free insulin-like growth factors (IGFs), and adipocyte-derived factors that include leptin, tumor necrosis factor-alpha (TNF-α), interleukin (IL) 6, and reductions in adiponectin [5]. Several theories have been proposed to explain the association between elevated BMI and postoperative complications. One potential explanation suggests that obese patients face a higher risk for surgical site infections after open surgery. Wound infection in obese patients might be related to the presence of excessive fat tissue, which has low regional oxygen tension, and therefore might predispose to impaired wound healing and infection in the presence of concomitant factors such as hematoma [7]. Another possible mechanism is that obesity causes alterations in the respiratory system, leading to a higher risk of perioperative pulmonary complications [10]. Additionally, obesity is often accompanied by comorbidities such as hypertension (HTN), diabetes, and cardiovascular disease, which may contribute to an increased risk of postoperative complications [11]. Given the conflicting evidence regarding the relationship between BMI and postoperative complications in colorectal cancer patients, further investigation is needed.

This proposed study aims to address this gap by conducting a retrospective cohort analysis of patients undergoing surgical resection for colorectal cancer. This study primarily investigates whether a higher BMI, the presence of comorbidities, tumor characteristics, and the type of surgical intervention increase the likelihood of postoperative complications such as wound infections, pulmonary complications, anastomotic leak, venous thromboembolism (VTE), bowel obstruction, and incisional hernia. In addition to the primary aim, this study describes the characteristics of colorectal cancer patients with different BMI groups including patients’ demographics, American Society of Anesthesiologists (ASA) grade, length of hospital stay (LOS), and development of postoperative complications.

## Materials and methods

The study was conducted in King Abdulaziz Medical City (KAMC) in Riyadh, a tertiary National Guard Hospital instituted in May 1983, with a capacity of 3,133 beds approximately. This study included all patients aged 18 years old or older who underwent elective or emergency surgery for colorectal cancer between the years 2015 and 2022. it is a retrospective cohort study with non-probability consecutive sampling, by including all patients who meet the inclusion criteria.

Prior to conducting the study, ethical approval was obtained from the Institutional Review Board of King Abdullah International Medical Research Center (KAIMRC) (approval number: IRB/1598/23).

Data was collected by chart review using the BESTcare system (Saudi-Korean Health Informatics Company, Riyadh, Saudi Arabia) at KAMC. Only the research team members collected the data. Informed consent was not required because it is a retrospective cohort study. All data was kept safely, and no identification data was asked such as medical record number (MRN), names, and ID (MRN replaced with serial number). Subjects’ privacy and confidentiality were assured, no identifiers were collected, and all data was kept in a secure place within National Guard Health Affairs (NGHA) premises, both hard and soft copies.

The collected data included patient’s demographics such as age, gender, and body mass index (BMI); patient’s comorbidities such as hypertension, diabetes, dyslipidemia, benign prostatic hyperplasia, hypothyroidism, and asthma; and other variables such as the American Society of Anesthesiologists (ASA) grade, surgery type, peri- and postoperative complications, tumor location and tumor-node-metastasis (TNM) stage, hemoglobin (Hgb) and albumin levels, and if the patient is deceased or alive.

Statistical Package for Social Sciences (SPSS) version 27 (IBM SPSS Statistics, Armonk, NY) was used. Mean and standard deviation (SD) were used for quantitative variables. Qualitative variables were expressed as percentages and frequencies. Non-parametric tests were used to compare the variables that were not following the normal distribution curve. Quantitative variables were compared between groups using the analysis of variance (ANOVA) test. A p-value of 0.05 will be considered significant.

## Results

The study is a retrospective cohort investigation that screened a total of 122 individuals diagnosed with colorectal cancer at a tertiary National Guard Hospital located in Riyadh, Saudi Arabia. The primary aim of this research was to evaluate the relationship between elevated body mass index (BMI) and the occurrence of postoperative complications among individuals who underwent surgical resection for colorectal cancer. A thorough analysis of descriptive data was performed to assess the correlation between high BMI levels and the likelihood of experiencing postoperative complications, as well as the duration of hospital stay. Additionally, the study explored the potential associations between coexisting comorbidities and tumor characteristics with the odds of experiencing postoperative complications.

As outlined in Table 1, a total of 122 patients diagnosed with colorectal cancer were included in the study. These cases were categorized into six different body mass index (BMI) groups. Among them, a single male case (0.8%) fell under the underweight category (BMI of <18.5 kg/m²), while 29 (23.8%) had a normal weight (BMI of ≥18.5 to <25 kg/m²), 49 (40.2%) were classified as overweight (BMI of ≥25 to <30 kg/m²), and 43 (35.3%) were categorized as obese (BMI of ≥30 kg/m²). Within the obese group, there were subgroups: obese I (BMI of ≥30 to <34.9 kg/m²), obese II (BMI of ≥35 to <39.9 kg/m²), and obese III (BMI of ≥40 kg/m²), accounting for 21 (17.2%), 13 (10.7%), and nine (7.4%) of the total participants, respectively.

**Table 1:** Characteristics of colorectal cancer patients with different BMI (n = 122) ^a^ANOVA test; ^b, c, e, f^Fisher exact test; ^g^Kruskal-Wallis test ^1^Two cases were excluded (N/A); ^2^in males: 138-172 g/L and in females: 121-151 g/L; ^3^normal albumin = 34-54 g/L; ^4^interquartile range A p-value of less than 0.05 is considered significant BMI, body mass index; SD, standard deviation; ASA, American Society of Anesthesiologists; Hgb, hemoglobin; ANOVA, analysis of variance; N/A, not applicable

	BMI						P-value
	Underweight, n (%)	Normal weight, n (%)	Overweight, n (%)	Obese (I), n (%)	Obese (II), n (%)	Obese (III), n (%)	
	1 (0.8)	29 (23.8)	49 (40.2)	21 (17.2)	13 (10.7)	9 (7.4)	
Age (years) mean ± SD (64.18 ± 13.25)	68.00	62.97 ± 14.69	63.47 ± 11.98	65 ± 15.04	61.62 ± 13.22	72.67 ± 10.29	0.448^a^
Gender							
Male (n = 79)	1 (0.8)	23 (79.3)	35 (71.4)	12 (57.1)	7 (53.8)	1 (11.1)	0.004^b^
Female (n = 43)	0 (0.0)	6 (20.7)	14 (28.6)	9 (42.9)	6 (46.2)	8 (88.2)	
ASA classification^1^							
ASA-1 (n = 5)	0 (0.0)	3 (10.4)	1 (2.0)	0 (0.0)	1 (7.7)	0 (0.0)	0.188^c^
ASA-2 (n = 35)	0 (0.0)	8 (27.5)	20 (40.8)	3 (14.3)	3 (23.0)	2 (22.3)	
ASA-3 (n = 73)	1 (0.8)	18 (62.1)	23 (47.0)	16 (76.2)	8 (61.6)	7 (77.7)	
ASA-4 (n = 7)	0 (0.0)	0 (0.0)	4 (8.2)	2 (9.5)	1 (7.7)	0 (0.0)	
Hgb level (g/L)							
Normal Hgb^2^ (n = 31)	0 (0.0)	4 (13.8)	20 (40.8)	3 (14.3)	1 (7.7)	3 (33.4)	0.026^d^
Low Hgb (n = 91)	1	25 (86.2)	29 (59.2)	18 (85.7)	12 (92.3)	6 (66.6)	
Albumin level (g/L)							
Normal albumin level^3^ (n = 80)	0 (0.0)	20 (69.0)	34 (69.4)	13 (61.9)	9 (69.2)	4 (44.5)	0.348^e^
Hypoalbuminemia (n = 41)	1 (0.8)	9 (31.0)	15 (30.6)	8 (38.1)	4 (30.8)	4 (44.5)	
Hyper-albuminemia (n = 1)	0 (0.0)	0 (0.0)	0 (0.0)	0 (0.0)	0 (0.0)	1 (11.0)	
Postoperative complications							
Yes (n = 33)	1 (0.8)	8 (27.6)	14 (28.6)	1 (4.8)	5 (38.5)	4 (44.5)	0.036^f^
No (n = 89)	0 (0.0)	21 (72.4)	35 (71.4)	20 (95.2)	8 (61.5)	5 (55.5)	
	Median (IQR^4^)						
Length of hospital stay (days)	0	8 (6-12)	8 (7-12)	8 (6.5-12)	7 (6-11)	11 (7.5-16.5)	0.414^g^

The average age was 64.18 years, with a standard deviation (SD) of 13.25. Of the participants, nearly two-thirds (n = 79, 64.8%) were male, while the remaining individuals were female. Laboratory tests indicated that just below three-quarters of cases (n = 91, 74.6%) had hemoglobin (Hgb) levels below the standard range for both genders. Almost one-third of the total patients (n = 41, 33.6%) had albumin levels below the standard level too. The American Society of Anesthesiologists (ASA) physical status scores were applied to all cancer patients, revealing that only five (4.1%) individuals were classified as physically healthy (ASA-1), whereas 117 (95.9%) exhibited various severity degrees of systemic disease, ranging from mild systemic disease (ASA-2) in 35 (28.7%) patients to severe systemic disease limiting daily activities (ASA-3) in 73 (59.8%) patients.

Additionally, seven (5.7%) patients were classified as having severe systemic disease that was incapacitating (ASA-4). Following the surgical resection of colorectal tumors, 33 (27%) patients experienced postoperative complications, with an average hospital stay duration of 9.67 ± 3.98 days, and no cases of 30-day mortality were recorded.

The study findings showed no statistically significant difference among different ages in relation to various ranges of BMI scores (p-value > 0.05). Similarly, being categorized within any BMI scale range did not impact hospital stay duration, ASA scores, or blood albumin levels (p-value > 0.05). Conversely, the study identified statistically significant correlations among different BMI categories for gender, blood hemoglobin levels, and the incidence of postoperative complications (p-value < 0.05 for all). Additionally, males had higher BMI scores compared to females. Notably, patients with elevated BMI levels experienced a significant reduction in hemoglobin levels when compared to those with normal BMI scores. A statistically significant association was found between increased BMI scores and the incidence of postoperative complications (p-value < 0.05). Out of the patients who experienced postoperative complications (n = 33, 27%), 24 (19.7%) patients fell in BMI score category of ≥25 kg/m².

As shown in Table 2, the majority of patients diagnosed with colorectal cancer (n = 89, 73.0%) did not encounter postoperative complications. Among the group with complications (n = 33, 27.0%), venous thromboembolism (VTE) resulting from extended immobility was identified in six (4.9%) cases, while postoperative fluid accumulation was observed in five (4.1%) cases. Additionally, an array of complications were noted, encompassing arrhythmia, bowel obstruction, diarrhea, hematoma, incisional hernia, lower urinary tract symptoms, pneumonia, and postoperative ileus, each presenting in two (1.6%) cases. The remaining six complications, namely, anastomotic leak, anemia, bleeding per rectum, perineal abscess, urinary tract infection, and wound site infection, were each observed in a singular (0.8%) case. No death incidence was reported in the study sample indicated by the (0%) 30-day mortality.

**Table 2:** Prevalence of postoperative complications among colorectal cancer patients (n = 122)

Postoperative complications	n (%)
Anastomotic leak	1 (0.8)
Arrhythmia	2 (1.6)
Bowel obstruction	2 (1.6)
Diarrhea	2 (1.6)
Hematoma	2 (1.6)
Anemia	1 (0.8)
Incisional hernia	2 (1.6)
Lower urinary tract symptoms	2 (1.6)
Bleeding per rectum	1 (0.8)
Perinatal abscess	1 (0.8)
Pneumonia	2 (1.6)
Postoperative fluid collection	5 (4.1)
Postoperative ileus	2 (1.6)
Urinary tract infection	1 (0.8)
Venous thromboembolism	6 (4.9)
Wound site infection	1 (0.8)
None	89 (73.0)
30-day mortality	0 (0)

Table 3 shows postoperative complications in relation to BMI categories, comorbidities, and tumor characteristics. All comorbidities (HTN, diabetes mellitus {DM}, etc.) and TNM stage showed no statistical significance with p-value of 0.05. However, BMI categories, tumor location, and surgical intervention showed statistically significant association with p-value of <0.05. Calculated relative risk (RR) for most BMI classes, HTN, DM, asthma, and others indicates that postoperative complications are more likely to occur in these groups with RR of >1. Additionally, tumor located in the descending colon and rectosigmoid showed RR of >1, which indicates that postoperative complications are more likely to occur in these groups. Moreover, all TNM stages showed RR of >1, which indicates that complications are more likely to occur in all TNM stages.

**Table 3:** Postoperative complications in relation to BMI categories, comorbidities, and tumor characteristics A p-value of less than 0.05 is considered significant. An RR of more than 1 indicates that the event is more likely to occur BMI, body mass index; RR, relative risk; CI, confidence interval; DM, diabetes mellitus; BPH, benign prostatic hyperplasia; TNM, tumor-node-metastasis; N/A, not applicable

		Postoperative complication, n (%)		RR	95% CI	P-value
		No, n = 89 (72.95%)	Yes, n = 33 (27.04%)			
BMI categories						
Underweight (n = 1)		0 (0%)	1 (3.03%)	3.781	2.809-5.089	<0.0001
Normal (n = 29)		21 (23.59%)	8 (24.24%)	1.026	0.520-2.022	0.94
Overweight (n = 49)		35 (39.32%)	14 (42.42%)	1.097	0.609-1.976	0.755
Obese 1 (n = 21)		20 (22.47%)	1 (3.03%)	0.15	0.021-1.039	0.054
Obese 2 (n = 13)		8 (8.98%)	5 (15.15%)	1.497	0.701-3.195	0.296
Obese 3 (n = 9)		5 (5.6%)	4 (12.12%)	1.731	0.782-3.835	0.175
Comorbidities						
Hypertension	No (n = 52)	38 (42.7%)	14 (42.4%)	1.0082	0.559-1.818	0.978
	Yes (n = 70)	51 (57.3%)	19 (57.6%)			
DM	No (n = 66)	50 (56.2%)	16 (48.5%)	1.252	0.699-2.242	0.4491
	Yes (n = 56)	39 (43.8%)	17 (51.5%)			
Dyslipidemia	No (n = 78)	55 (61.8%)	23 (69.7%)	0.77	0.404-1.467	0.428
	Yes (n = 44)	34 (38.2%)	10 (30.3%)			
BPH	No (n = 108)	77 (86.5%)	31 (93.9%)	0.497	0.133-1.857	0.29
	Yes (n = 14)	12 (13.5%)	2 (6.1%)			
Hypothyroidism	No (n = 115)	83 (93.3%)	32 (97%)	0.513	0.081-3.227	0.477
	Yes (n = 7)	6 (6.7%)	1 (3%)			
Asthma	No (n = 111)	82 (92.2%)	29 (87.8%)	1.391	0.599-3.230	0.441
	Yes (n = 11)	7 (7.8%)	4 (12.2%)			
History of malignancy	No (n = 119)	86 (96.6%)	33 (100%)	0.447	0.033-6.081	0.546
	Yes (n = 3)	3 (3.4%)	0 (0%)			
Others	No (n = 63)	46 (51.7%)	17 (51.5%)	1.005	0.561-1.800	0.986
	Yes (n = 59)	43 (48.3%)	16 (48.5%)			
Tumor location						
Ascending colon	No (n = 90)	62 (69.7%)	28 (84.8%)	0.502	0.212-1.189	0.117
	Yes (n = 32)	27 (30.3%)	5 (15.2%)			
Descending colon	No (n = 115)	85 (95.5%)	30 (90.9%)	1.642	0.661-4.077	0.284
	Yes (n = 7)	4 (4.5%)	3 (9.1%)			
Transverse colon	No (n = 105)	73 (82%)	32 (97%)	0.193	0.028-1.320	0.093
	Yes (n = 17)	16 (18%)	1 (3%)			
Rectosigmoid	No (n = 56)	47 (52.8%)	9 (27.3%)	2.262	1.148-4.458	0.0183
	Yes (n = 66)	42 (47.2%)	24 (72.7%)			
TNM stage						
I and II	No (n = 79)	59 (66.3%)	20 (60.6%)	1.194	0.66-2.157	0.556
	Yes (n = 43)	30 (33.7%)	13 (39.4%)			
III and IV	No (n = 62)	46 (51.7%)	16 (48.5%)	1.097	0.612-1.967	0.753
	Yes (n = 60)	43 (48.3%)	17 (51.5%)			
Not staged	(n = 19)	16 (18%)	3 (9.1%)	N/A	N/A	N/A
Surgical intervention						
Partial resection (n = 120)	Yes (n = 120)	89 (100%)	31 (93.93%)	0.258	0.190-0.349	0.013
Total resection (n = 2)	Yes (n = 2)	0 (0%)	2 (6.06%)	3.871	2.858-5.241	

## Discussion

Colorectal cancer ranks among the most prevalent types of cancer, and surgical intervention is one of the treatments of choice. Several postoperative complications commonly arise; however, the timely and proper management of these complications can dramatically reduce postoperative morbidity and mortality rates [12]. A high body mass index (BMI), including overweight (BMI of ≥25-29.9 kg/m²) or obesity (BMI of ≥30 kg/m²), has been associated with unfavorable outcomes in cancer, such as higher recurrence and mortality rates. Probable reasons, including reduced treatment sensitivity, could potentially account for these adverse results observed in patients with obesity [13]. In the Arab world, colorectal cancer (CRC) stands as a big concern. A comprehensive literature review study conducted by Makhlouf et al. (2021) revealed a substantial prevalence of CRC in numerous Arabic nations, including Saudi Arabia, Egypt, Qatar, and the United Arab Emirates [14].

In this study, most colorectal cancer (CRC) patients (n = 89, 73%) with no complications following their operative procedures were found to have lower body mass index (BMI), if compared with those who did experience postoperative complications (n = 33, 27%). Upon the analysis of the relative risk (RR) between the BMI group and the development of postoperative complications, the findings suggest that having obesity class 2 or 3 may be associated with the increased risk of postoperative complications; on the other hand, having obesity class 1 showed a lower risk. However, it is important to keep in mind that this study is based on a small sample size. This finding suggests that the outcomes of CRC surgery are primarily influenced by factors other than excess body weight, such as tumor stage, demographic variables (e.g., age and gender), and various preoperative and intraoperative care measures. Excess body weight was reported to only contribute to the progression of postoperative wound infections [13,15,16].

Within this cohort sample, no reported cases of mortality within 30 days of surgery were documented. The mean duration of postoperative hospitalization for the study group was 9.67 ± 3.98. Notably, there were no statistically significant differences in the length of hospital stay (LOS) across various BMI categories (p-value > 0.05). In this respect, the relationship between BMI and LOS showed inconsistencies across different studies. While some studies indicated that surgically treated patients with higher BMI scores exhibited lower mortality rates and shorter postoperative hospital stays, others identified higher mortality rates and prolonged hospitalization periods [17-20]. These variations could likely be attributed to differing factors, such as sociodemographic, clinical, and financial.

No significant differences were observed among various age groups across all BMI categories (p-value > 0.05) in this study. However, male patients exhibited higher BMI scores compared to their female counterparts (p-value = 0.004). Similarly, Hauck and Hollingsworth (2010) reported a higher number of males with elevated BMI scores compared to females, which aligns with this study findings [21]. This observation corresponds with the study conducted by Bardou et al. (2013), revealing that male patients with higher BMI values were more susceptible to colorectal cancer than their female counterparts [13].

No statistically significant association was observed between the ASA physical status of cancer patients and BMI categories (p-value = 0.188). However, Arkenbosch et al. (2019) documented a significant variation in ASA scores across the four subgroups of BMI [22]. Similarly, this study did not reveal any significant link between various comorbidities such as hypertension, diabetes, hypothyroidism, and the occurrence of postoperative complications (p-value > 0.05). This contradicts other research studies that highlighted a strong association between preexisting systemic diseases, particularly cardiopulmonary conditions, and an increased risk of severe complications following surgery [16].

Furthermore, this study found no substantial correlation between tumor characteristics and the emergence of postoperative complications, except for tumor location and the type of surgery performed (p-value < 0.05). Notably, tumors situated in the rectosigmoid or rectum regions and patients who underwent total resection exhibited a higher risk of postoperative complications (RR > 1 and 95% confidence interval {CI}: 2.858-5.241). However, partial resection was associated with less risk of developing postoperative complications (RR < 1 and 95% CI: 0.190-0.349). In alignment with this study findings, Law et al. (2007) also concluded that emergency procedures rather than elective operations and patients with rectal cancer presented with significantly higher complication rates after surgery. These complications, in turn, impact overall survival and could contribute to an elevated recurrence rate [23].

Retrospective cohort studies using medical records have key limitations to consider. Data accuracy cannot be affirmed due to errors and missing or unclear data. Selection bias also might be misleading, affecting the representation of certain cases. Confounding variables could impact results, and causality cannot be definitively established. Findings are limited to this study sample and could not be generalized. Unmeasured variables and the lack of control over data collection are also concerns. Despite these, such studies offer practical insights into medical practices and outcomes.

## Conclusions

In conclusion, this study highlights the impact of BMI on postoperative outcomes in colorectal cancer patients. Higher BMI was associated with unfavorable postoperative outcomes, such as an increased risk of VTE and fluid collection. However, no significant differences in mortality rates or length of hospital stay were observed across various BMI categories. Factors beyond BMI, including tumor characteristics, the type of surgical intervention, and preoperative care, play a significant role in determining postoperative outcomes. Therefore, it is essential to adopt a comprehensive approach that considers multiple factors when managing postoperative complications in colorectal cancer patients, particularly those with higher BMI.
